# Community and cultural engagement for people with lived experience of mental health conditions: what are the barriers and enablers?

**DOI:** 10.1186/s40359-022-00775-y

**Published:** 2022-03-16

**Authors:** Louise Baxter, Alexandra Burton, Daisy Fancourt

**Affiliations:** 1 Department of Behavioural Science and Health, University College London, London, UK; 2 Present Address: Department of Social Sciences and Social Work, Bournemouth University, Bournemouth, UK

**Keywords:** Mental health, Community and cultural engagement, Qualitative

## Abstract

**Background:**

Community and cultural engagement can support recovery, help symptom management and increase social connections for people with lived experience of mental health conditions. However, research suggests that people with mental health conditions experience significant barriers to participation. The aim of this study was to explore barriers and enablers of participation in community and cultural activities among people with mental health conditions.

**Methods:**

A qualitative interview study with 23 people with mild-to-moderate mental health conditions was undertaken. Data were analysed thematically, and themes were mapped to domains of the Capability, Opportunity and Motivation Model of Behaviour (COM-B).

**Results:**

Eleven themes were identified from the analysis. Three themes involved participant Capability: physical skills, psychological traits and physical health limitations and three themes related to Opportunity: affordability and accessibility, structure and nature of the group, and support from others to attend. Five themes mapped to Motivation: creative identity, recovery and coping, enjoyment and fun, connecting with others, and information and planning. Participants were motivated to engage with community and cultural activities through “a creative identity”, belief that engagement would help recovery from mental illness, and a desire to connect with others and make friends. Motivation to participate was sustained by the enjoyable nature of activities. However, participants’ ability to engage was hampered by the expense, inaccessibility and sometimes unstructured nature of activities, and social anxiety associated with attending. Some participants had physical limitations such as fatigue or physical health problems to overcome. Interventions that could address these barriers include peer support, training for social prescribers to account for identity and previous experiences of participation, training for community organisations in providing a welcoming and structured environment, and provision of long-term sustainable funding to community organisations to subsidise attendance, transport or equipment costs.

**Conclusion:**

People with mental health conditions may be at risk of experiencing barriers to community and cultural engagement due to existing social inequalities and social anxiety, however believing that involvement will support mental health was an enabler to participation. Future studies are needed to test the effectiveness of potential interventions to address the barriers and harness the facilitators identified here, to enable a more socially inclusive community and voluntary sector, and a potentially more responsive and effective social prescribing service in the UK for people experiencing mental health problems.

**Supplementary Information:**

The online version contains supplementary material available at 10.1186/s40359-022-00775-y.

## Background

People with lived experience of a mental health condition often experience social exclusion through reduced social participation in their communities [1, 2]. In turn this reduced social contact and support are recognised as risk factors for exacerbating mental ill-health [1, 3, 4]. Community and cultural engagement (CCE) in activities such as community arts, volunteering or social groups can address some of these issues through supporting recovery, helping people to cope with symptoms, or by increasing social networks [5–7]. For example, there is evidence that participatory arts projects can significantly improve mental health and reduce social exclusion [5, 8, 9] and improve wellbeing [10, 11]. Access to, and use of green space such as parks and gardens has been found to support mental health and improve symptoms [12, 13], whilst volunteering can reduce depression and improve life satisfaction [14].

Despite growing evidence that CCE can improve mental health, there is concomitant evidence suggesting significant barriers to participation for people with lived experience of mental illness. Barriers and enablers of participation can be conceptualised using the COM-B behaviour change framework, which posits that behaviour (B) is influenced by Capability [C], Opportunity [O] and Motivation [M] [15]. Further, capability to perform a behaviour can be understood as either physical (e.g. skill or strength) or psychological (e.g. knowledge), while opportunity can be broken down into either physical (e.g. environment and resource) or social (e.g. cultural norms, social support or cues). Finally, motivation can be reflective (e.g. planning and evaluating) or automatic (impulses and desires). People with low happiness have been found to engage less in cultural activities than people with higher happiness levels [16]. This difference is partly explained by education and socio-economic status (elements of physical capability and opportunity) and a perceived lack of psychological capability and social opportunity [17]. However, given that low levels of happiness (part of the broader construct of wellbeing) is a related but distinct concept from mental illness, it remains unclear whether these results can be extrapolated to explain barriers to engagement for people with mental illnesses.

Few qualitative studies have explored specific barriers to, or enablers of, CCE amongst people living with mental illnesses. Fear of being patronised (an element of reflective motivation) may act as a barrier to engaging in museum activities [18], whilst access to transport (physical opportunity), social support networks (social opportunity), and the building of coping strategies and creative identities (reflective and automatic motivation) have been found to act as enablers [6, 19–21]. However, to date there have been no studies that systematically and comprehensively explore barriers and enablers to CCE amongst people living with mental illnesses.

In light of the growing evidence for the benefits of CCE, the National Health Service (NHS) in England and Wales has, since 2019, recruited approximately 1,200 workers to implement ‘Social Prescribing’ in primary care, with the aim of reaching 900,000 people by 2023/24 [22]. Social prescribing aims to link patients to community and cultural groups, and to services such as job centres, to help address increasing demand for psychosocial support in primary care and to reduce social isolation [23]. However, there is evidence that uptake of and adherence to such interventions is low [24]. This, combined with evidence that there could be barriers to participation specific to people with mental illnesses, suggests a need to find solutions that reduce inequalities in participation and to ensure effective implementation of schemes such as Social Prescribing to improve mental health. Therefore, this study aimed to identify barriers and enablers to CCE among individuals with lived experience of mild-to-moderate mental illnesses with varying levels of self-directed involvement in community and cultural activities.

## Methods

### Design

Qualitative interviews were used to explore barriers and enablers to CCE among people with lived experience of mental illnesses. The study took a phenomenological approach [25], focusing on participants lived experiences of what helped or hindered their engagement in community and cultural activities. We defined CCE as engaging with the arts, culture and heritage, libraries and literature, sports and nature activities, volunteering, or community groups (see Table 1).

**Table 1:** Examples of cultural and community engagement (CCE)

Type of CCE	Example activities
Arts	Performing art groups (e.g. music/dance/theatre); visual arts and crafts groups (e.g. textiles/drawing/woodwork/painting/photography/ceramics/sculpture)
Culture and heritage	Museums, galleries, exhibitions; theatre, concerts; cinema; festivals, fairs, events; stately homes/buildings; historical sites; landscapes of significance
Libraries and literature	Libraries; visiting archives; book clubs, writing groups
Sports and nature activities	Visiting parks/gardens; allotments or gardening groups; nature walks or rambling groups; exercise classes; sports club membership; community sporting activity participation; attending amateur/professional sporting events
Volunteering	Charitable/conservation volunteering, school/community volunteering
Community groups	Education/evening classes; political, trade union or environmental groups; tenant/resident/neighbourhood watch groups; social clubs

One-to-one, semi-structured interviews were conducted by LB: a female postdoctoral researcher with expertise in mental health research and qualitative methodology, either in person at the researcher’s place of work, at the participant’s home, within community organisations or, to increase geographical coverage of the study, via online video-call. Interviews took place between May 2019 and March 2020 and lasted between 35 and 70 min. Data collection continued until theoretical saturation [26], whereby no new concepts pertaining to barriers and facilitators for CCE were discussed by interviewees.

### Participants & procedure

A purposive sampling approach was taken [27] to reflect potential differences in barriers and enablers attributable to level of participation or group/activity type. Recruitment took place via emails to member organisations of the MARCH Mental Health Research Network (https://marchlegacy.org/) which is comprised of community organisations that provide art activities, volunteering, or social support to people with mental health problems. LB also visited community mental health support groups to promote the study in person, and information about the study was shared on social media (Facebook and Twitter), and via the MQ research organisation participant recruitment tool: https://participate.mqmentalhealth.org/ which specifically advertises research opportunities to people experiencing mental health problems. Inclusion criteria were: aged 18 + , self-reporting a mild or moderate mental health condition and sufficient understanding of English to provide informed consent and participate in an interview. We specifically focused on a broad sample of people with self-reported mild-to-moderate mental illness for three reasons: (i) many mental health problems are not formally reported, and we did not want to limit our sample only to people in contact with professional services who had received a formal diagnosis; (ii) as there has been very little research into barriers or enablers of CCE amongst people living with mental illness, we took a broad approach for this first study, with the aim that the findings here could be elaborated by more specific studies of particular mental illnesses in the future; and (iii) this study was designed with a secondary aim of providing evidence that could be of use to the cultural and community sectors in making their activities more “mental health friendly” – an endeavour that would need to be relevant to people with a broad range of mental health experiences.

Thirty-seven people expressed an interest in taking part in the study and were sent the participant information sheet. 14 people declined to be interviewed or did not respond further to the invitation. Twenty-three people participated in the study. Travel expenses were reimbursed if participants travelled to the interview. No other incentives were offered. The study received ethical approval from the University College London (UCL) ethics committee (14895/001). All participants provided written informed consent.

An interview topic guide was developed, informed by the COM-B model (Additional file 1: Appendix; Topic Guide: People with lived experience of mental illness). Interviews were audio-recorded and transcribed by an external company (www.waywithwords.net) in anonymised form.

### Data analysis

The analytical approach was ‘reflexive thematic analysis’ [26, 28] and involved familiarisation with the data, generating initial codes, searching for themes, reviewing themes (codes and themes were discussed and agreed by two researchers – LB and DF: associate professor in psychobiology and epidemiology with expertise in social and community engagement and mental health), defining and naming themes, and producing the report. A combination of inductive and deductive approaches was implemented: initial coding was undertaken in an inductive and open manner to allow for the codes and themes to be grounded within the data. Coding was undertaken by LB using NVivo software (Version 12). Contradictory data were included and context around codes was retained. Coding therefore included not only what was explicitly expressed by participants (description) but was also interpreted to comprehend the barriers and enablers to participation within the framework of the COM-B model.

Codes were grouped into sub-themes and then assigned to overarching themes which in turn represent a central definition [28]. The themes were then mapped to the three domains of the COM-B model: Capability, Opportunity and Motivation, and identified as barriers, enablers, or both.

## Results

CCE among participants ranged from none, or some past participation but none in the present, to extensive participation across multiple activities. Active participants took part in a range of activities, including choirs, dancing, volunteering and cultural engagement (e.g. performance or gallery attendance). Participants all self-reported having a mild or moderate mental health condition. While the researchers did not directly ask for information on specific mental health conditions, 19 participants made reference to their mental health conditions during the interview (See Table 2 for participant demographics).

**Table 2:** Participant demographics

Participant characteristics	Number in study
Gender	
Male	8
Female	15
Age	
20–29	2
30–39	5
40–49	6
50–59	4
60–69	1
70–79	3
Not disclosed	2
Experiences of mental health conditions	
Anxiety	6
Depression	6
Anxiety & depression/low mood	4
Not disclosed	4
Eating disorder	1
Bipolar disorder	1
Bipolar disorder, depression, anxiety & PTSD	1
Region	
Wales	1
London	8
East England	3
West England	2
North England	1
South England	3
Midlands	3
Not disclosed	2

### Themes

Eleven themes were identified and grouped by the COM-B model domains: Capability, Opportunity and Motivation.. Five themes mapped to Motivation: Creative and community identity, Recovery and coping, Enjoyment and fun, Connecting with others, and Information and planning. Within Opportunity, there were three themes: Affordability and accessibility, Structure and nature of the group, and Support from others to attend. Three themes mapped to Capability: Physical skills, Psychological traits, and Physical health limitations.

**Fig. 1:**
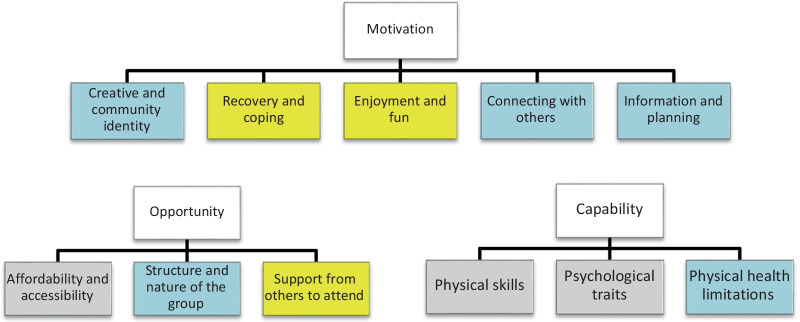
Themes grouped by COM-B model domains (Yellow denotes enabler, Grey: barrier, Blue: both)

Sub-themes, and COM-B model domains are outlined in Fig. 1.

### Motivation

The desire to nurture or develop a community and creative identity, often instilled in childhood was highly motivating, although this could be experienced as a barrier to involvement where opportunities did not match participant interests. Participants reported that CCE could help them manage depression or anxiety and that this was key to their initial motivation to participate; with enjoyment helping to maintain involvement. Wanting to make friends and socialise was a positive motivator, but social anxiety was a barrier to engagement.

### Creative and community identity

Identifying as a creative or community-focused person was a key motivation for CCE. This identity often originated in childhood:

I was just looking to do a hobby and something to get me out of the house, and I think just because I did it as a child. (*Participant_14*).

Positive experiences in childhood could lead to revisiting the activity in adulthood: *“I think okay I’ve done this before, and I’ve managed okay.” (Participant_5)* and helped motivate people to use CCE deliberately to support their mental health:

I went to another school and this was around the time I started to engage with music, because I found something that started to make my brain work differently …I just latched onto this thing that I feel completely changed me...So, I think, without realising it, it was a complete game changer in my life. (*Participant_8*).

Positive experiences of CCE with family members in childhood were important in fostering a sense of CCE as fun and enjoyable:

I was brought up in an environment where art was quite important. We were taken to museums and stuff. So…music and art were always part of our time to enjoy (*Participant_2*).

This perception of a creative and community identity could also work to define who a participant was *not.* For example, adverse childhood experiences, such as negative feedback from teachers or bullying around physical appearance, acted as barriers to participation in certain types of CCE in adulthood. This forming of an identity has implications for the types of activities people with lived experience of mental illness might consider, or reject, when suggested by health professionals:

Well partly because I used to get picked on a lot at primary school…I was the kid who got picked last for rounders and things. I got bullied by a teacher I had at primary school for not being very good at...I couldn't hit the ball *(Participant_3).*

Previous negative experiences could also be used as motivating factors when participants decided that they wished to ‘turn their lives around’ and try something new to aid recovery:

I thought, well I can’t sing so I won't be able to do that. So, I didn't choose it. And I think, looking back, it’s something I really regret. I think that was one of the motivations for joining the choir. Just to do it and see what happened (*Participant_3*).

Sometimes participants were reluctant to identify as creative because of the perceived need to possess a high level of skill:

Do I dare to claim this identity and this level of mastery? You know, you don’t have to be particularly good at it to be a dancer, you can just do it. But it still felt like me claiming…[I have] a bit of imposter syndrome about it (*Participant_5*).

There were participants who did not express a creative identity initially, but for whom the development of identity through CCE led to a ‘virtuous circle’ of involvement and helpful experiences:

I think before it would be more like, I dabble in…dancing, or I’ve been taking lessons for a few months or something, or oh I dance sometimes but I’m rubbish. Now a combination of getting better at it and also the amount of time it takes up in my life, I’m like, all right, I surrender, I’m a dancer. (*Participant_5*).

### Recovery and coping

Most participants expressed a belief that participation would help them to recover, or cope with mental illness. Repeated participation was recognised as important for the benefits of the activity to be felt, and beliefs about these benefits could help overcome reluctance to engage:

I call it my Oyster Card *(smart card that you add money to, so you can pay for travel as you go).* I top it up and it gets me through the next week. Sometimes you don’t want to go but you go because you know the benefits (*Participant_4*).

CCE helped participants to manage their mental illness by giving a structure to the day, not only attending the activity, but also planning the day around it: *“it gives me structure to mean that I have to go and talk to people, I have to go do something” (Participant_17)*. A desire to be active, and to be involved in their communities, or in cultural activities was often expressed.

Participants also expressed a desire to have *“achieved something” (Participant_13).* CCE offered a supportive way to try something new and was perceived as helpful for managing depression and anxiety. This in turn was linked to a desire to participate to overcome stigma towards themselves, and others:

It does break down the stigma because, when we sang at <concert hall> with what we call the posh choirs…We sang with the <Name> Community Choir. And they had difficulty with a few of us that…A couple that weren’t very well on the day. But the next time we met, there was a great difference of understanding (*Participant_4).*

Participants who had positive experiences of CCE were often involved in multiple activities or had taken on increased responsibilities such as committee or trustee posts, suggesting another type of ‘virtuous circle’ of motivation, whereby initial involvement helped with recovery or coping strategies, thus enabling further participation.

For some, the activity represented a new start, a ‘break’ from their illness, and a chance to build a new aspect of their identity:

And I thought, okay, this is my new life or whatever and I’m going to go ahead and try this (*Participant_5*).

### Enjoyment and fun

Enjoyment and fun were important motivators for participation in community activities. Enjoyment cut across different ‘types’ of activity, from attending theatre performances to visiting parks. This was crucial for continued engagement:

It’s joyful and I love it. I love doing things I like and the nice people. It is, it’s really uplifting. It’s really a joyful experience, something I wouldn’t miss unless I had to do something else. (*Participant_10*).

Enjoyment was enhanced by the lack of pressure or need for particular skills when initiating activities. One participant enjoyed dance classes where the moves required were “*goofy*”, another referred to how much she laughed during choir sessions. Several found that the lack of pressure was concomitant with enjoyable activities, and this in turn encouraged sustained engagement:

It’s talking and being with people but no pressure to be perfect. So, that’s why I enjoy it. (*Participant_4*).

Enjoyment was attributed to the skills of group facilitators*: “I think because of the teacher, she just makes it fun” (Participant_14)* and to the shared enjoyment of group activities: *“you go just for the enjoyment of seeing people, having a good sing.” (Participant_4).* This enjoyment contributed to prolonged participation in deeper ways, participants described having an “*emotional break*” from the reality of their lives and responsibilities. In this way the activity contributed to the development, or reinforcing of a valued creative identity:

It keeps my finger on the pulse. It keeps me in touch with myself. It’s what defines who I am as a person. It helps give me that emotional break from the responsibilities I have. It really feeds my soul to do these things. (*Participant_20*).

### Connecting with others

The desire to make friends was a significant motivator for initiating CCE: *“I want to meet other people who I can learn from…yes, to meet new people and make new friends” (Participant_22)* and friendships also motivated participants to sustain participation:

if you’re lucky, you make friends with them. And it gives me a routine, gets me out of the house and I get some company there, that’s why really (*Participant_13*).

This connection extended beyond personal friendships to a ‘sense of community’. The strength of relationships developed from community participation influenced other significant life decisions for one participant:

I’ve thought about just for general life reasons moving elsewhere in Europe and I think part of the reason I haven’t…is because I really like the dance community here and I have so many friends here (*Participant_5*).

Feeling included was a central aspect of connecting with others. One participant missed the ‘family feel’ of an organisation that had experienced budget cuts:

that was the difference…there was more staff and they had talks where everybody was included that they don’t have now (*Participant_13*).

Volunteering also provided a team of ‘colleagues’ for those who didn’t work. One participant volunteering across several roles commented:

I really like the group of people I work with and we’ve been working together for several years (*Participant_10*).

A need for interactions in person, rather than online was described for personal relationships to develop:

I have tried online book clubs and things like this, but what I’ve found is that it’s not real interaction or it’s not the same kind of interaction…So, the fact that you compose a message and you send it off, and then somebody else sends one back, it doesn’t create a real, personal relationship at all (*Participant_16*).

Several participants however, described their experience of mental illness as a hinderance to initial participation, usually due to low confidence and social anxiety:

It’s one thing…to find out about it off the internet or through word of mouth or through a poster. But to actually then get to the next stage, which is the participation stage, that’s a very complex hurdle to overcome if you’re lacking social confidence (*Participant_1*).

This was especially acute for those not currently engaging in community or cultural activities:

I’m a very people person. Even if I’m not feeling up to it, I will go out, but I’ve never been in this mental state before. But, it’s the first time I’m feeling safer in my home than to step out. So, what will stop me today, a good example is my anxiety’s extremely high and, at times, on the verge of panicking (*Participant_20*).

Dislike of groups, anxiety about how the participant would be perceived and a fear of rejection by the group were also identified as barriers to attendance. This was often linked to negative experiences of groups in childhood:

I think it just takes me back to childhood where I was that introverted kid and I felt like I stuck out like a sore thumb, because people tend to make friends easily, but I don’t. And so …I avoid doing stuff like that now because I just imagine it is going to be like what I was as a child (*Participant_14*).

### Information and planning

Accessible information was an enabler to engagement, either helping those already motivated to find initial opportunities: *“If I’m interested, I see an advert and I think, that looks really interesting”* (*Participant_18*) or supporting continued engagement: a newsletter reminded one participant that cultural events were happening in her community.

However, many participants described a lack of accessible information about activities and events, particularly from grassroots and local groups without mailing lists or advertising campaigns. Groups with pages on social media platforms such as Facebook increased their ‘discoverability’, but this was a barrier if participants were not linked into such platforms:

Maybe because I don’t know Facebook well enough, I don’t know how to discover, by geography, groups who don’t have a public website. Because, obviously, if you don’t have a public website, you don’t appear on Google searches, either. So, if you don’t already know about these groups, I don’t know how to find them (*Participant_16*).

### Opportunity

Affordability and accessibility of activities represented a significant barrier to participation. The ‘structure and nature’ of groups comprised a mixture of barriers and enablers: how the group was organised, whether the facilitator was trusted, and the inclusive (or otherwise) nature of the group. Active support from family, friends or peer supporters (e.g. providing transport, or attending with the participant) could be a strong enabler, but support from health professionals was less powerful.

### Affordability and accessibility

Being unable to afford participation was a barrier for most participants. This was a paradox for those advised to take part in activities for their mental health but unable to afford them:

I lost my job through mental illness. I have had to rely on the dreaded universal credit. So, there is a lot I can’t do. For example, I have been told I need to do yoga and pilates and stuff like that. And I literally can’t afford it. (*Participant_12*).

Costs could also lead to a valued activity being curtailed: *“That was the reason I gave up after the year, just because I couldn’t afford it anymore” (Participant 14).* Even free activities such as volunteering were prohibitively expensive in equipment costs, thereby potentially excluding people with lived experience of mental illness from these roles: *“that can play itself out in terms of even just having the travel money and being able to buy lunch to having the right clothing and equipment if that’s needed.” (Participant_1).* This could further prevent involvement through concern about not having *the “right kit, the right stuff”* which in turn lead to *“social fear” (Participant_1).*

Being offered free or subsidised activities was an enabler to participation. One participant who described money as “constantly a problem” was taking part in “*a free term of ballet for beginners which I had done before as a beginner in my very early twenties” (Participant_3).* This allowed them to choose activities which fit their interests and were therefore more likely to be sustained.

A lack of available activities did not appear to be a barrier to CCE (although lack of information about activities was a barrier to motivation, as discussed above). However, participants did struggle with a lack of public transport to access activities and anxieties about using public transport:

I don’t drive either, so if there’s then anything that I’m like, oh, that sounds quite good, it’s a drive away. And then I look at public transport and I just can’t get there *(Participant_14)*.

Participants emphasised the importance of local activities:

For me in [location], it was just the next town over, so it’s a 20 minute drive, yes that was an attractor as well, finding somewhere local (*Participant_21*).

The lack or expense of using public transport was particularly problematic in rural communities:

And, after the class, I would be too tired. It wouldn’t be safe to drive. So, you need someone who can drive you. So, you have to either force someone else to come along… Taxi would be £25, there and back. So, that’s too expensive (*Participant_6*).

However, it was acknowledged that community and cultural activities were sometimes more accessible than conventional health services:

[Referring to the rural surrounding area] It’s a pain if you want to access services because there’s none on the ground. I think that’s why art group is so important. Because a lot of people can’t access any services so they come along to [the group]. (*Participant_6*).

Activity schedules also significantly impacted engagement. Medication side effects were identified as a barrier, with participants often unable to attend morning activities. For one participant, the group facilitator helped to mitigate this:

He started up the group. And it was in the afternoon, because I can’t get out of bed early in the morning, most of the time, because of the medication…I really wanted to go (*Participant_19*).

Work commitments also presented a barrier to participation. Participants who were well enough to work found that they were less likely to find time to engage in community activities. Groups and classes are often scheduled during the day, with those working full time unable to attend, even when motivated to do so:

I was trying to find something that I could do in the creative arts and I was looking at the adult education, because I work, most of the adult education stuff is in the day. It’s like 11 to three, and there was nothing I could do (*Participant_7*).

When evening or weekend activities were available, participants often felt too tired to attend:

When I’m in work and working really hard, it just takes my mind off my situation. And then I’ll tend to just get home really late and then just get something to eat and then go to bed, and then it’s another day done with (*Participant_14*).

Caring responsibilities were a further barrier to participation. One participant described the impact of new caring responsibilities on her previously high levels of community involvement:

So, the reason I’m not engaged is because I think I’m just trying to find myself a little bit. I’m trying to find my feet (*Participant_20*).

Both types of responsibility could intersect with the experience of mental illness to compound these problems. Participants were often frustrated; able to see how involvement could help them manage the stresses of work or caring, but unable to access activities due to these commitments:

I know full well there’s a load of things that I’ve never been exposed to that I might find a lot of pleasure in, but I’m not doing it because I can’t get my head around it. How am I going to find the time and organise myself to do that? And that in itself is very damaging because then you start getting really quite annoyed and thinking, well, where do I go from here? (*Participant_8*).

### Structure and nature of the group

Most participants spoke about looking for groups with ‘structure’ including how the group was run, or groups that had a specific purpose, such as a choir or dance class:

I like it because it is structured and if you do want to stand to the side and talk to people more and stuff, you can do that. But you can also just dance (*Participant_5*).

A lack of structure was cited as a reason to avoid participation:

Certainly for the first time it’s getting over that hump of this is a weird, unstructured social thing where I probably have to small talk and mingle, and that’s… Particularly if I’m feeling anxious or going through a bad patch with depression, just no. (*Participant_5*).

One participant liked to socialise, but it was the structured opportunity to do so that would enable participation:

The difference between me being able to do things that you might think would cause anxiety and things that do cause me some anxiety, I think is structure (*Participant_16*).

Facilitators who were perceived as helpful and supportive were also central enablers to participation:

My tutor makes all of this possible by trying to help me… to try to do better than my head says (*Participant_17*).

Most participants found the tendency of groups to meet in the pub a key barrier to participation. Many did not like the conflation of the group with drinking, or the way that this could lead to a less structured social group: “*I don’t drink and I’m not comfortable in a pub atmosphere*”:

So, yes, the pub-style socialising, both for work and for these out of work groups, I feel uncomfortable with (*Participant_16*).

The interaction of alcohol with medication or experiences of poor mental health often meant being unable to drink, and presented a further barrier when activities were organised around drinking:

There’s a Knit and Natter in a knitting shop, but it also involves drinking gin. Now, that’s not going to really work for somebody who’s on medication, who can’t drink, because they’re automatically not going to be able to really join in. (*Participant_11*).

Participants expressed different preferences around attending groups explicitly for people with lived experience of mental illness. Some were concerned that this would put strain on their mental health:

When you are feeling unwell and you are trying to recover even more, I think being with people who are not necessarily always very well is a bit difficult on your own mental health (Participant_2).

For some, this felt stigmatizing: *“it implies that there’s something wrong with you if you go to that” (Participant_22).* However, for others, participating with people with similar experiences was important: *“I think it probably is a bit more appealing, because they could probably understand more” (Participant_9).* For some, this was linked to wanting to attend groups with “*people like me*”.

A key attraction of activities was the flexibility in socialising with others, particularly where the activity was aimed at people with lived experience of mental illness:

You don’t have to talk to people if you don’t want to, and I found that that was helpful depending on how I felt. I said to people, look, if I’m not talking to you, it’s not because I don’t want to, it’s just what’s going on with me. And they were fine with that. (*Participant_17*).

Whether the group felt inclusive and welcoming were essential for continued involvement:

It was lovely. It was really welcoming, and she was really sweet. And straight away, because of [a fellow group member] I felt quite at ease (*Participant_2*).

This welcoming environment from peers was linked to feeling ‘no pressure’ from the group which was a significant factor in continued enjoyment of the activity:

And there’s no pressure so it’s not even like being in the church choir. There’s no pressure. You don’t have to hit a note. So, what’s what I enjoyed. Having a good belt at a song, it does make you feel good… there’s no pressure at all (*Participant_4*).

### Support from others to attend

Attending activities with family or friends was an enabler of engagement. Whilst some mentioned being *“willing to go…if somebody else in my circle goes” (Participant_20*) it was important that this was active rather than passive support: *“she’s the one who’s more likely to book tickets and then ring me and say, I’ve booked us tickets, let’s go to the theatre” (Participant_20).* Several participants mentioned siblings or friends that attended classes with them. Siblings were often mentioned as more outgoing, providing access to groups that participants felt they could not attend or settle into by themselves:

Yes. I do struggle to go when my sister isn’t there. She was on holiday for three weeks at the start of the year and…I really had to force myself to go (*Participant_14*).

Not everyone wanted family or friends to be involved, especially where the activity enabled individual participation: *“You don’t have to go with somebody. You don’t have to be supported to go” (Participant_21).* This was an exceptional viewpoint, however; most found it *“a lot easier if you go with someone else” (Participant_16).*

Whilst active support from family and friends was important for engagement for some participants, support from family or friends may not be available to all. Peer support was felt by many to be crucial to increased CCE, from the point at which an individual decided to participate in activities, through to encouraging continued attendance:

There was this one girl…and she would sit next to me and she would talk…she would make me relax…if she hadn’t have been there, I would have started crying. And I don’t know whether I would have left the room or what (*Participant_19*).

Participants who did not currently engage in community and cultural activities described needing sustained “practical and emotional” support to attend:

If somebody would come and meet me, several times, at home. And then take me…I’d be more inclined to go. But they’d need to come and meet me several times, so I have confidence in them. And then they’d have to come and take me and bring me back. And stay with me while I was there (*Participant_20*).

Peer support for CCE was also identified as a missing element of the social prescribing pathway:

The difficulty, currently, with the way social prescribing is set up, is there’s no support to get to the place. They say, do this, do that. But if you are anxious or you don’t want to meet new people or are worried about meeting, you need somebody to go with you. (*Participant_21*).

Most participants had not received advice from health professionals to engage in cultural or community activities to support their mental health*: “no, I haven’t had that kind of advice”* (*Participant_16*). Where this advice was received, there was often a delay between receiving the advice and engagement. One interviewee suggested that this was because her doctor had suggested gardening which didn’t match her interests or creative identity.

### Capability

Capability for CCE appeared to be less of a barrier than ‘motivation’ or ‘opportunity’ however some participants described needing to overcome common physical symptoms that accompany mental illness such as fatigue. ‘Physical skills’ encompassed both low perceived abilities, and the presence or absence of ‘actual skills’. ‘Psychological traits’ such as lack of confidence and social skills were also barriers to participation.

### Physical skills

Despite high levels of motivation for involvement in community activities, some participants, particularly those with limited current engagement, perceived a lack of skills and ability to engage, particularly in arts, crafts or dancing. This was linked to fears that others in the group would be highly skilled:

The skills thing too…I don’t really know how to knit, will anybody be there who would be happy to teach me? I don’t want to distract people from their projects. And probably if I could knit a bit, I’d be like, oh but my knitting is terrible (*Participant_5*).

Beyond the *perception* of feeling unskilled, or unable to learn new skills, some participants described an absence of actual skills as preventing participation in activities they were interested in. Participants however expressed a willingness to learn and often participated to develop new skills, further demonstrating that physical capability was not always an important barrier to involvement:

I play at home with clay. I don’t know if it’s good or not. But I’m proud of what I do. I’m like, I love that something, whatever this is. So, I join just to learn more about technique and stuff. So, I don’t think skills is something that would be a barrier. (*Participant_15*).

Participants were also able to use other skills to overcome a lack of ‘hard’ skills in order to learn:

So yes, the ability to laugh at yourself and to be patient with yourself, and the ability I guess to forgive yourself for messing up or whatever (*Participant_17*).

### Psychological traits

Psychological traits were more commonly identified as barriers, such as a lack of confidence when participating in groups:

Lack of confidence with new people. I get very anxious when I’m in a new social situation. I’m quite quiet. I don’t speak up because I feel nervous. And that’s why I’m so tired on the way home (*Participant_6*).

Lack of confidence could hinder involvement by limiting learning opportunities, or crucially, the opportunity for connection with others:

I don’t have certain kinds of social skills and I don’t have confidence in those skills. But those things are the difference between, I feel, being able to socialise and finding it difficult to socialise (*Participant_16*).

This need for “social effort” was felt to be a difficult barrier to traverse, particularly for those who were not currently participating in activities:

It’s not exactly fear…It’s just like, you know what? It’s too much effort to get everything ironed and go there and try and be nice…you know you’ve got to smile at everybody all the time (*Participant_14*).

Not every participant lacked social confidence and skills. For example, one commented that:

I’ve never felt like I’ve needed the skill to do the things that I’ve done. I’ve just gone and done it because I’m quite outgoing (*Participant_20*).

However, increasing self-confidence was identified as a welcome outcome of CCE, and confidence to engage in groups was often increased by sustained involvement:

These two years of doing public workshops, I did maybe three or four a year, and then gave me the confidence to do the [longer]…so it’s helped me achieve much more than I dreamed (*Participant_7*).

### Physical health limitations

Some participants reported challenges in overcoming physical health limitations to take part including chronic illnesses, medication side effects, or physical symptoms that accompany mental illness:

Stamina…that is something that is impacted by my depression and anxiety. Sometimes I just don’t feel like I have the energy (*Participant_5*).

Physical health limitations led some to select different activities (e.g. singing instead of gardening: “*I’d love to do gardening but, obviously, there’s no way, physically, that I’d be able to” (Participant_2)* but no participants named physical health limitations as a reason to not participate at all. Instead, strategies were employed to address a ‘bad’ day:

My mental health has impacted me, particularly if I’m having bouts of insomnia, so ideally, I come home from work, and maybe I sort out whatever life admin things I have to do, and then I go out to class. Sometimes if I’m really tired…then either I might skip class or usually I do multiple classes in one night when I do them, so I might just go to a later class (*Participant_5*).

For some, participation in group physical activities helped alleviate symptoms of poor mental health linked to physical health problems:

I’ve got some arthritis in my knees…I can walk for miles, I just can’t go fast. And then I do ache a bit afterwards, but I feel quite divine (*Participant_4*).

## Discussion

This study aimed to identity barriers and enablers of CCE amongst individuals with lived experience of mental illness. Enablers of engagement included peer support, belief that involvement would help individuals cope with mental illness, and the desire to connect with others and make friends. Barriers included affordability, accessibility, social anxiety, poor physical health and lack of opportunities that matched creative interests and identities. Given the strong evidence for the mental health benefits of CCE, identifying these barriers and enablers is an important step for ensuring access amongst individuals who stand to benefit most from engagement.

Interventions are needed to address these barriers so that people with experience of mental illness can fully participate in CCE. It is recommended that interventions seeking to address behaviour change at an individual level are designed using a system that matches appropriate interventions to features of the target population, the context, and the specific barriers identified [15]. The behaviour change wheel (BCW) situates the COM-B model at its centre, around which are placed different intervention functions that seek to address barriers in one or more of these concepts [29]. Mapping the COM-B barriers identified in this study onto the BCW highlights several types of interventions that could encourage and enable participation by people with lived experience of mental illness, which are outlined below.

### Motivation

Many participants had positive childhood experiences of the arts, and these experiences influenced motivation to participate, or which activity to participate in. This was linked to perceived ‘artistic’ identity in childhood that, whilst a positive influence on participation, also guided participants away from certain activities. The importance of childhood participation and ‘artistic’ identity has been reported in healthy samples too [35] and was found not only to predict attendance at activities, but also improved wellbeing [36]. Previous qualitative work suggests that participation in community or cultural activities allows participants to develop a ‘renewed identity’ associated with creativity and being an ‘artist’ in contrast to an identify associated with mental ill-health [6, 7]. Participants in this study who identified as ‘artistic’ recognised that this expression helped them manage symptoms and recovery. Having a creative identity in childhood has been found to allow people with mental illness to ‘return’ to these former identities to help with building new ones [6]. These findings have an important implication for practice: it is important for social prescribing link workers to understand the patient holistically, and gain an understanding of identity, interests and previous experiences of the arts for suggested activities to be acceptable and therefore successful. A further policy implication is that of creative education and opportunities for young people: the development of early positive relationships with community and cultural activities may pave the way for improved mental health, and thus improve the effectiveness of those tools in secondary prevention for people with mental ill health.

Connecting with others and making friends were also important in enabling CCE behaviours, however, many participants felt held back by social anxiety. High levels of ‘perceived public stigma’ (how others view and treat you) have been reported by people with mental illness, particularly when seeking treatment [37]. Other social factors also related to opportunities to engage. Many participants felt hampered by a combined lack of social confidence, and the social structure of the groups, such as the organisation of social opportunities around a ‘pub’ or drinking culture. Participants were motivated to participate by clearly defined activities, directed group facilitation, and fixed times for group socialising, and were discouraged by a lack of structure. “Welcoming environments” have been previously identified as important enablers to participation [19, 21] and the additional desire for structured activities and avoiding drinking and pub culture carries implications for third sector organisations providing inclusive activities beyond those that are more commonly perceived as conducive to socialising. Participants were strongly in favour of ‘peer support’ to help increase CCE. Previous studies have shown benefits of peer support for mental health [38, 39] making it an important consideration in schemes such as social prescribing, where the link worker can help identify suitable activities that can be done with others. This suggests that the link worker role could be expanded to peer support workers or volunteers. Importantly, peer support should be available until participation is established.

### Opportunity

Affordability and accessibility of activities, how the groups were run, and support to attend were described by participants as a barrier to CCE. For many, particularly those in remote locations, public transport was a barrier to participation, and the attendant cost of getting to activities, especially if participants could not drive. While these barriers have been reported in healthy samples in previous studies [30], people with mental ill-health are more likely to be socio-economically disadvantaged [31, 32], so may experience this barrier more acutely than people without mental illness. Poverty or financial hardship also prevents social participation, which in turn can lead to social exclusion [1]. This extends to transport or purchasing equipment for activities. These financial difficulties are often compounded by being unable to work or access benefits. Participants in this study sometimes experienced a ‘double-bind’; when they were able to work, they were unable to continue CCE due to tiredness, or incompatible activity timetables. These barriers are predominantly structural and require a restructuring of the social and physical environment [15, 29]. Previous work has also found similar barriers to accessing community based physical activities among immigrant women (financial insecurity, transportation) as well as additional barriers (not feeling entitled to participate, language difficulties) [33]. Interventions therefore need to be designed in collaboration with people experiencing financial difficulties from different sociocultural backgrounds so that they can be tailored to their specific needs. As well as being an intervention to enhance motivation, peer support schemes could also tackle barriers related to opportunity to support CCE from the initial decision to attend (or as part of the link worker recommendation) through to establishment within a group or activity. Other potential interventions include funding for community and voluntary sector organisations to allow subsidised activities and transportation and evening activities, or training of link workers to ensure that signposting accounts for creative (or other) values or identities.

### Capability

Participants largely reported having the necessary psychological capabilities for CCE and selected activities based on past experiences, current interests, or to develop new skills. This finding echoes wider literature suggesting that barriers to CCE do not tend to relate to psychological traits or capabilities [6, 19, 21]. Physical limitations such as physical symptoms, chronic illness, or medication side effects, however, were a significant issue for many participants. Whilst recent work has been undertaken to make community and cultural activities disability friendly in light of some of the same barriers being reported by people with physical health conditions or disabilities, [34] further interventions that support enablement (e.g. adaptations to the environment where activities take place or the timetabling of events) are needed to ensure that activities are inclusive for people with lived experience of mental illness both in providing a welcoming environment and supporting people with comorbidities.

In considering how to encourage CCE, three factors stood out as important: believing in the benefits of CCE, identity, and social connections. Almost all participants believed that participation in some form of activity would help them to overcome, or cope with symptoms of mental illness, and this in part drove engagement. Even those not currently engaged in community activities recognised the likely positive impact of doing so, and most wished to return to or become involved in the future. Perceiving that participation in creative and community activities will help symptoms of mental ill-health has been found in similar studies [19, 21], and evidence for actual participation helping to achieve better mental health also exists [5, 8]. Therefore, education interventions that increase knowledge of the benefits of CCE, such as leaflets provided by GPs alongside social prescribing referrals, could be of value.

### Strengths and limitations

Participants reported varied levels of cultural and community participation and engaged in a wide range of activities, allowing the barriers and enablers experienced by participants across the sector to be more deeply understood. Interviews were offered online, as well as in person, depending on participant preferences, which allowed a greater geographical range of participants to be included, as well as flexibility on interview times. The research was guided by an established theoretical framework, allowing us to ground our suggestions for intervention components in the data. However, there were some limitations. Participants primarily experienced mild to moderate depression or anxiety, and it may be that more severe mental illnesses present different barriers to participation. We did not ask participants specific details of their diagnosis as this study was focused broadly on mental health and CCE, but future studies may wish to explore whether there are further specific findings for specific populations such as people with social anxiety disorder. Similarly, we did not collect information on the sociocultural/socioeconomic background of participants, which may play a role in whether and how they perceive and approach participation in CCE. The relationship between our findings and the specific sociocultural/socioeconomic background of individuals should be explored in future work. Moreover, the self-selection of participants meant most were engaged to some degree in cultural and community activities (although we did stratify on level of engagement). Whilst some were nonetheless not currently engaged in cultural and community activities, an interest in this topic was likely still present as participation in the study was voluntary. This could explain why the most common barrier to cultural participation reported amongst general populations (not being interested) (30), was not reported here. Therefore although present in this study, the views of people with mild-to-moderate mental illness who do not participate at all may be under-represented. Additionally, we focused on CCE as a collective activity, but future studies may warrant exploration of the barriers and enablers of specific interventions or programmes to support scaling-up or rolling-out.

## Conclusions

This study explores the barriers and enablers to CCE for people with lived experience of mild-to-moderate mental illness. While some of these barriers and enablers have been reported in previous studies of healthy populations (including issues around affordability, accessibility), people with mental illness may be particularly at risk of facing such barriers due to existing social inequalities in mental illness. Further, other barriers such as social anxiety and enablers such as believing involvement will support mental health appear more specific to a lived experience of mental illness. A range of interventions could address these barriers, for example at the local level via peer support to encourage participation, and at the national level by training social prescribing link workers to consider a person’s identity and previous experiences of CCE when recommending activities. Future studies are encouraged that design, deliver and test the effectiveness of potential interventions that address the barriers and harness the facilitators identified here, to enable a more socially inclusive community and voluntary sector, and a potentially more responsive and effective social prescribing service in the UK.

## Supplementary Information

**Additional file 1. **Appendix: Topic Guide: People with lived experience of mental illness.

## Data Availability

The datasets and materials used and/or analysed during the current study are available from the corresponding author on reasonable request.

## Abbreviations

BCW: Behaviour change wheel
CCE: Community and cultural engagement
COM-B: Capability, opportunity and motivation model of behaviour
DF: Daisy fancourt
LB: Louise baxter
NHS: National health service
UCL: University college london
