# Supplementary material for: Community and cultural engagement for people with lived experience of mental health conditions: what are the barriers and enablers?
Source: BMC Psychol. 2022 Mar 16;10:71. doi: 10.1186/s40359-022-00775-y (PMC8928686; doi:10.1186/s40359-022-00775-y)
Supplement: Supplementary file 1 — Additional file 1. Appendix: Topic Guide: People with lived experience of mental illness. [file 40359_2022_775_MOESM1_ESM.docx]

**S1 Appendix Topic Guide: People with lived experience of mental illness**

Opening Question: both groups

**Please could you tell me about anything that you are involved in in your community at the moment? (Prompt with examples)**

***If not***

***Have you been involved in the past?***

*If yes:*

1. **Why do you take part in the activity? Do different factors influence the different activities you do?**

***Motivation prompts:***

1. ***When you first started doing the activity, what was it that motivated you to take it up? What is it that still motivates you to take part?***
2. *Is there anything you’re specifically hoping to achieve from taking part?*
3. *Are there any factors that have put you off from taking part?*
4. *would this be any different for any other kinds of activities*
5. *Do you find that taking part helps you with depression or anxiety?*
6. *Please tell us about this*
7. *Do you feel like a creative person/ gardener/ singer?*
8. *Did you always feel this way? Tell us about this*

***Opportunity prompts:***

1. ***What were the circumstances or opportunities that encouraged you to become involved?***
2. *Did you receive any advice or support about taking part in community activities?*
   1. *What was it?*
   2. *Did you act upon it? How?*
3. *Do you know anyone/ many other people who does/ do take part?*
   1. *Did they ask you to go along?*
   2. *Does their participation influence how you feel about taking part?*
4. *Have you ever had any worries about taking part due to not being able to access the activity or pay for lessons or equipment?*
5. *Have you ever had problems finding the time to take part in the activity?*

***Capability prompts:***

1. ***Do you think you have particular psychological or physical skills that support you in taking part in the activity?***
2. *Are there any other physical limitations or barriers to taking part in this activity?*
3. *Are there other activities that you would like to try, but are unable to because of the skills needed?*
4. *Do you feel you know how to do the activity well now?*
5. *Confidence around taking part in the activity? Did you feel confident in taking part in the activity before you started, or that you would have to learn a lot?*
6. *Did you find that you had to overcome your experiences of depression or anxiety in order to take part?*
7. **Do you ever find it hard to engage? Why?**
8. **Are there certain activities you wouldn’t want to engage in? Why?**

*If no:*

1. **Do you know what community assets are available to you?**
   1. **Please elaborate on these**
2. **What are the reasons for not becoming involved in any of these?**

***Motivation prompts:***

1. ***What might motivate you to take up an activity?***
   1. *What might motivate you to take up an activity?*
      1. *would this be any different for any other kinds of activities*
   2. *How do you feel about participating in community schemes, classes or groups? Have you ever wanted to take part in any of these? Why?*
      1. *Has this been the same for each of these?*
   3. *Are there any factors that have put you off from taking part? [make distinct from physical/ social resources]*
      1. *would this be any different for any other kinds of activities*
   4. *Is it important to you to take part in these activities? Why?*
      1. *would this be any different for any other kinds of activities*

***Opportunity prompts:***

1. **Have there been any circumstances that have put you off taking part?**
   1. *Have you ever received any advice or support from anyone or anywhere about taking part in schemes, classes or groups in your community?*
      1. *If yes, what kind of advice?*
      2. *Did you act upon it? How?*
   2. *Do you know anyone/ many other people who does/ do take part?*
      1. *Have they asked you to go along? Would this encourage you to do that?*
      2. *Does this influence how you feel about taking part?*
   3. *Are there any financial or logistical factors that stop you taking part?*
      1. *Please tell me how…For example, not having a car, money for classes, money for equipment, etc*
      2. *would this be any different for any other kinds of activities*
   4. *Do you think having more time to take part would help?*
      1. *Tell me about lack of time, or why not, etc…*
      2. *would this be any different for any other kinds of activities*

***Capability prompts:***

1. **Do you feel able to take part, do you have the skills to do this?**
   1. *Are there other physical limitations/ barriers to taking part in an activity?*
      1. *would this be any different for any other kinds of activities*

*b. Do you feel you know enough about the range of activities available? Is not knowing perhaps a barrier to taking part?*

*c. Do you feel your experiences of depression or anxiety hold you back from taking part?*
